# Supplementary material for: One Material-Opposite Triboelectrification: Molecular Engineering Regulated Triboelectrification on Silica Surface to Enhance TENG Efficiency
Source: Molecules. 2023 Jul 26;28(15):5662. doi: 10.3390/molecules28155662 (PMC10420044; doi:10.3390/molecules28155662)
Supplement: Supplementary file 1 [file molecules-28-05662-s001.zip › Suporting Information.pdf]

# *Supplementary Materials*

# One Material-Opposite Triboelectrification: Molecular Engineering Regulated Triboelectrification on Silica Surface to Enhance TENG Efficiency


**Mesude Zeliha Arkan [1], Zeynep Kinas [2], Eyup Yalcin [3], Emre Arkan [1,*], Faruk Ozel [4], Abdulkerim Karabiber [2] and Mirosław Chorążewski [1,*]**

[1] Institute of Chemistry, University of Silesia, Szkolna, 40-006 Katowice, Poland; mesude-zeliha.arkan@us.edu.pl

[2] Electrical Engineering Department, Bingol University, Bingol 12000, Türkiye; zeynepkns937@gmail.com (Z.K.); akarabiber@bingol.edu.tr (A.K.)

[3] Metallurgy and Materials Engineering Department, Ondokuz Mayis University, Samsun 55030, Türkiye; eyyup.yalcin@omu.edu.tr

[4] Department of Metallurgy and Materials Engineering, Karamanoglu Mehmetbey University, Karaman 70200, Türkiye; farukozel@kmu.edu.tr

* Correspondence: emre.arkan@us.edu.pl (E.A.); miroslaw.chorazewski@us.edu.pl (M.C.)


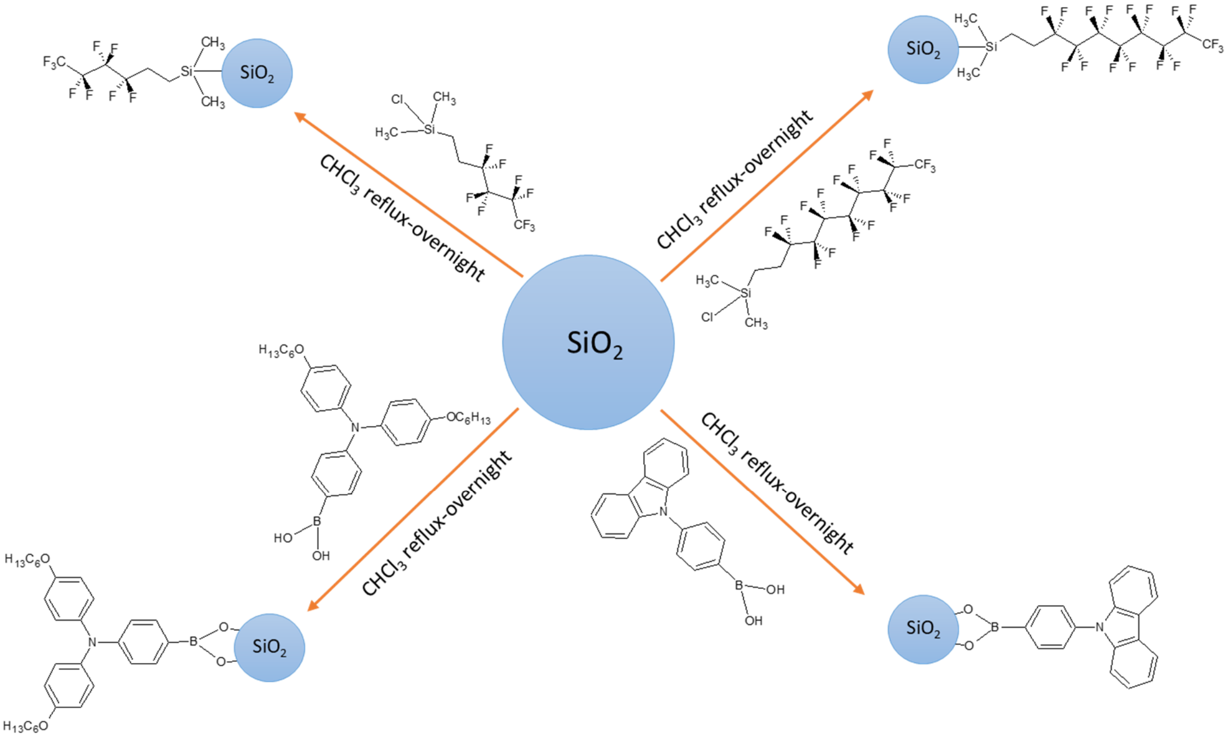


***Figure S1:*** *Synthesis diagram of ME hybrid structures.*

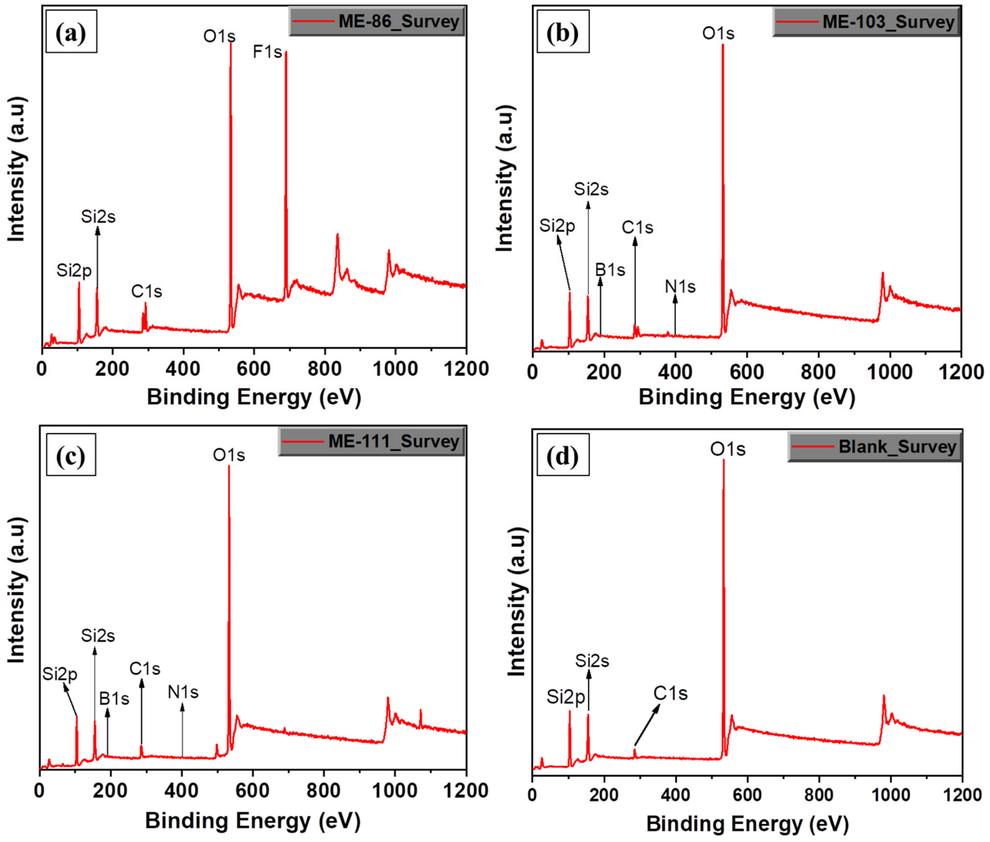


***Figure S2:*** *XPS survey spectrum of ME-86 (a), ME-103 (b), ME-111 (c), and bare -SiO₂ (d).*

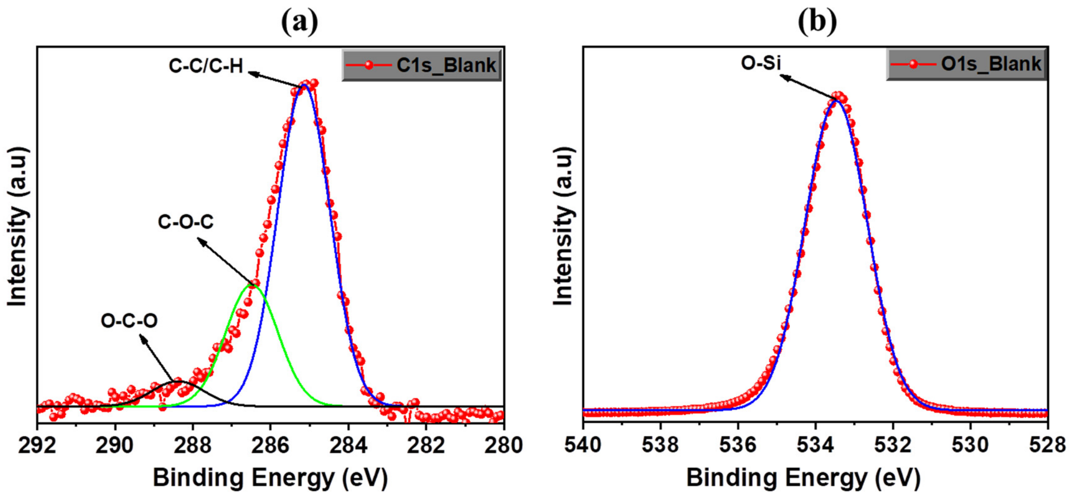


***Figure S3:*** *High-resolution survey spectrum of C1s (a) and O1s (b) for bare-SiO₂.*

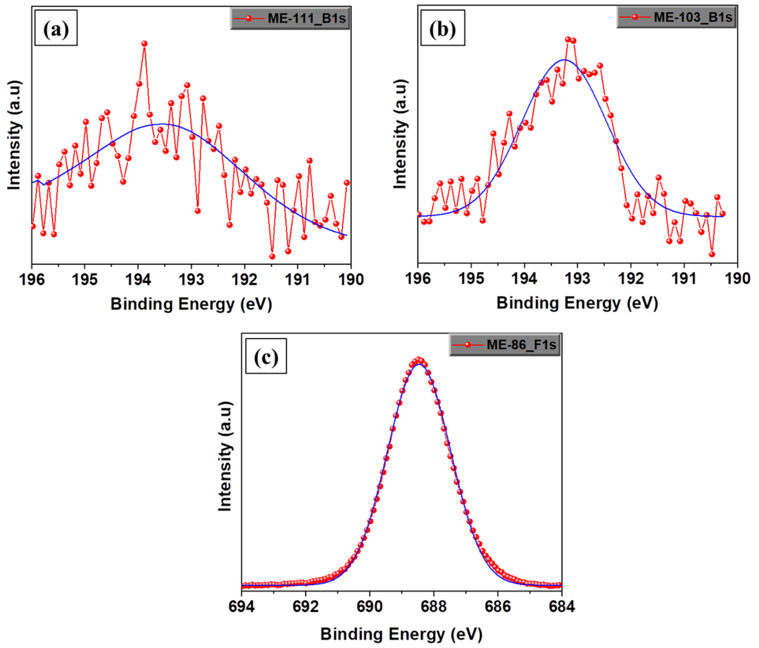


***Figure S4:*** *XPS high-resolution survey spectrum of B1s for ME-111 (a), ME-103 (b), and F1s for ME-86 (c).*

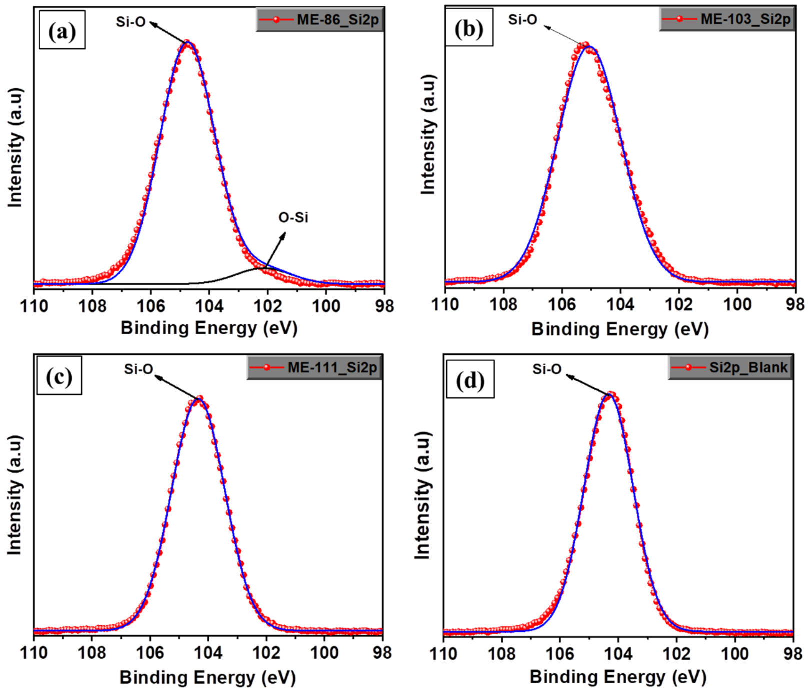


***Figure S5:*** *XPS high-resolution survey spectrum of Si2p for ME-86 (a), ME-103 (b), ME-111 (c), and bare-$SiO_2$ (d).*

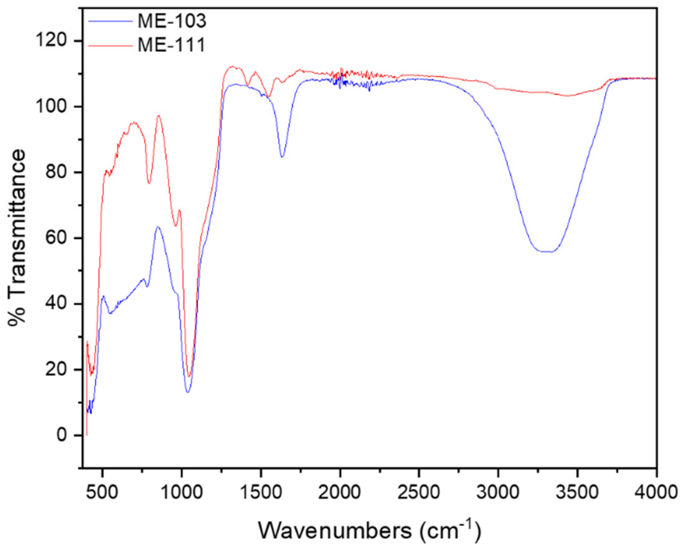


***Figure S6:*** *FT-IR analysis of ME-103 and ME-111 hybrid structures.*

Additional insight into the surface modification of silica by donor-organic moieties has been gathered by FT-IR analysis and obtained spectra are depicted in Fig.S1. The peak around 1020 cm-1 is attributed to the Si-O-Si stretching of the silica structure. While -OH peak is available in both ME-111 and ME-103, its existence is more dominant in the case of ME-103. This result may be explained by the fact that the bulky structure of the hexyloxy group on the organic modifier causes more steric hindrance that prevents the reaction on the silica surface and results in heterogeneity in surface modification and remaining the more silanols without substitution [1].

**ME-86/PAN**

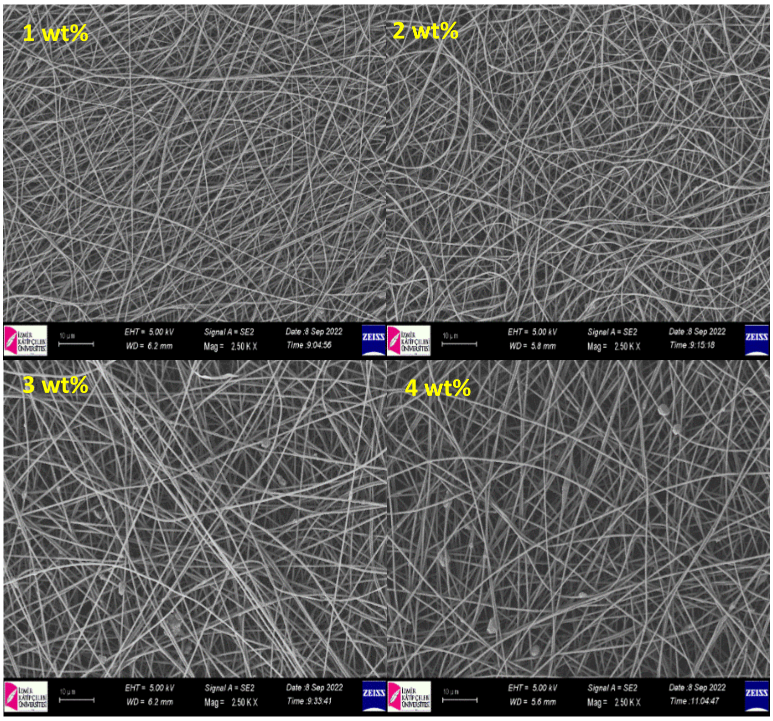


**ME-88/PAN**

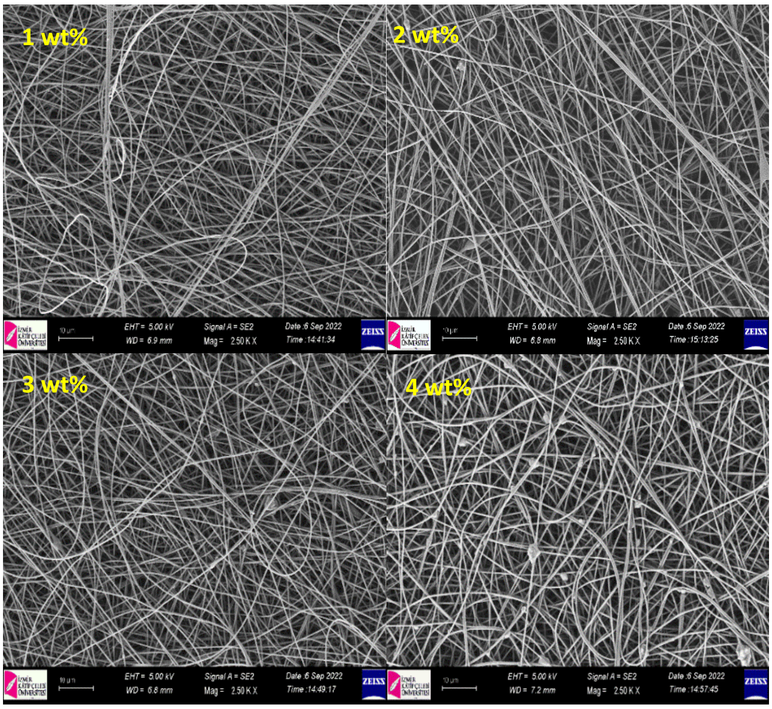


***Figure S7:*** *SEM images of ME-86 and ME-88 doped PAN fibers.*

**ME-86/ PAN**

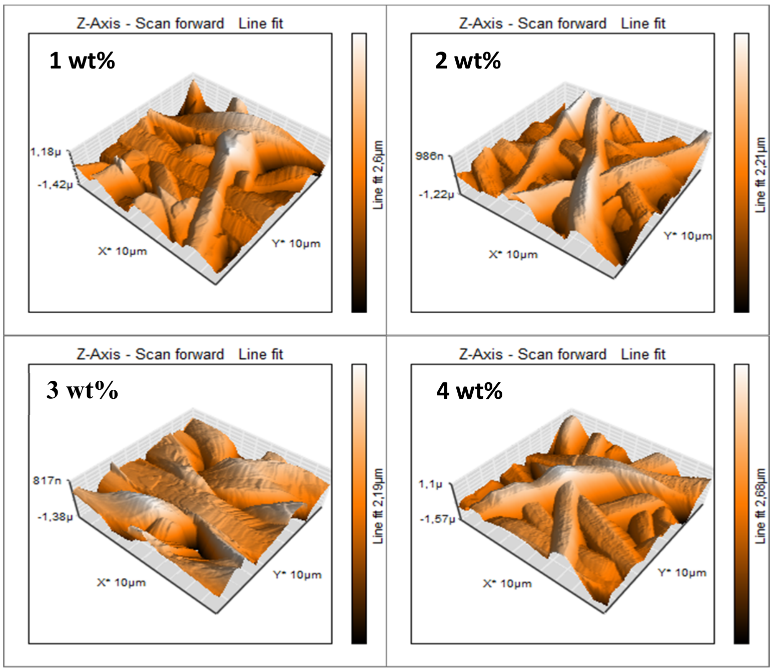


**ME-88 / PAN**

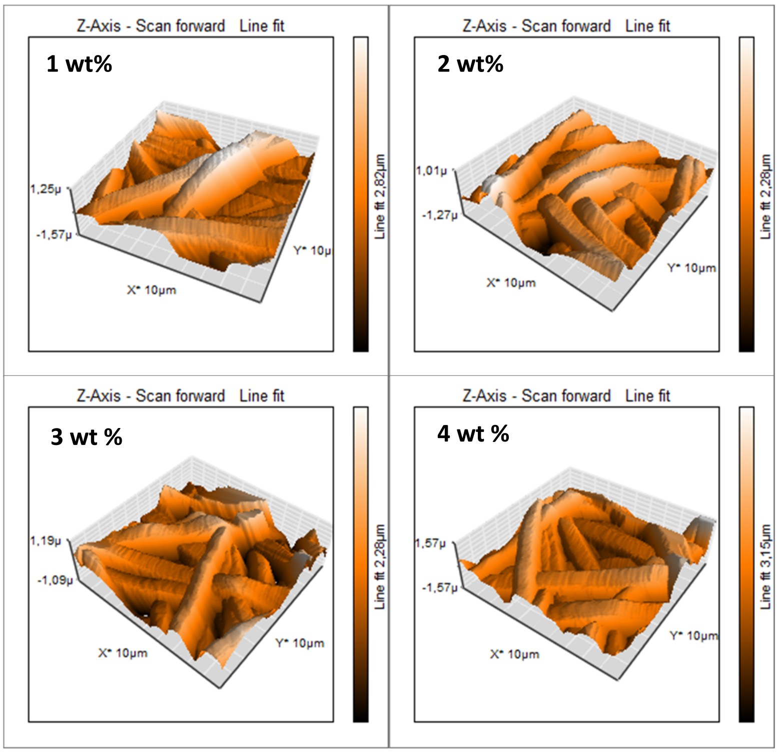


***Figure S8:*** *Topographical AFM images of ME-86 and ME-88 doped PAN fibers.*

**ME-86/ PAN** **ME-88 / PAN**

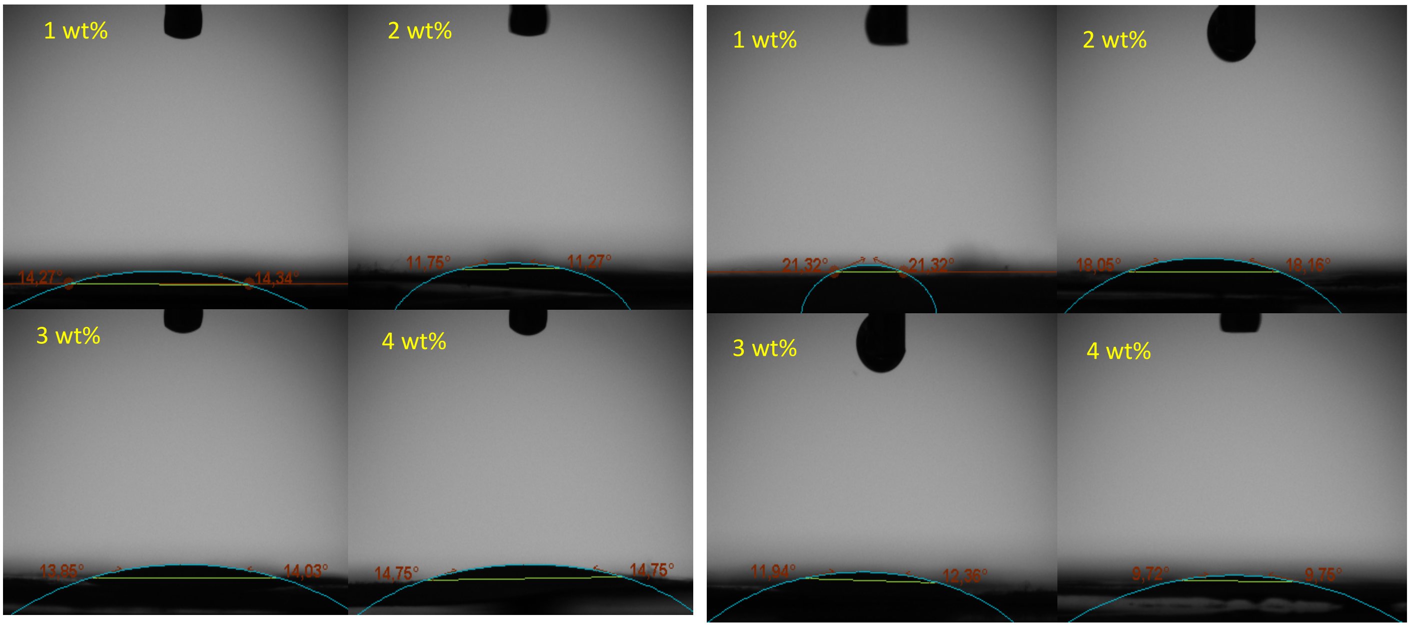


***Figure S9:*** *Contact Angle measurements of ME-86 and ME-88 doped PAN fibers.*

Since the fluorocarbon chains are inductive negative (-I), they not only have the ability to withdraw electrons towards themself but also a tendency to create hydrogen bonds with the hydrogen of PAN structure. This behavior increases the inequality in the electronic density of asymmetric molecules and enhances the electron density and polar interaction at the surface [2]. This dispersive interaction leads to an increase in surface energy and wettability of ME-86 and ME-88 doped polymers. Moreover, it is important to point out as an experimental observation that undoped PAN fiber films have less tightness, and therefore water droplets have quickly absorbed by the films. This is the reason why the water droplet in the image stayed as a small bead. By contrast, doping of ME-86, and ME-88 has increased the tightness of the fiber along with the wettability. So, water droplet has spread on the surface rather than being absorbed.

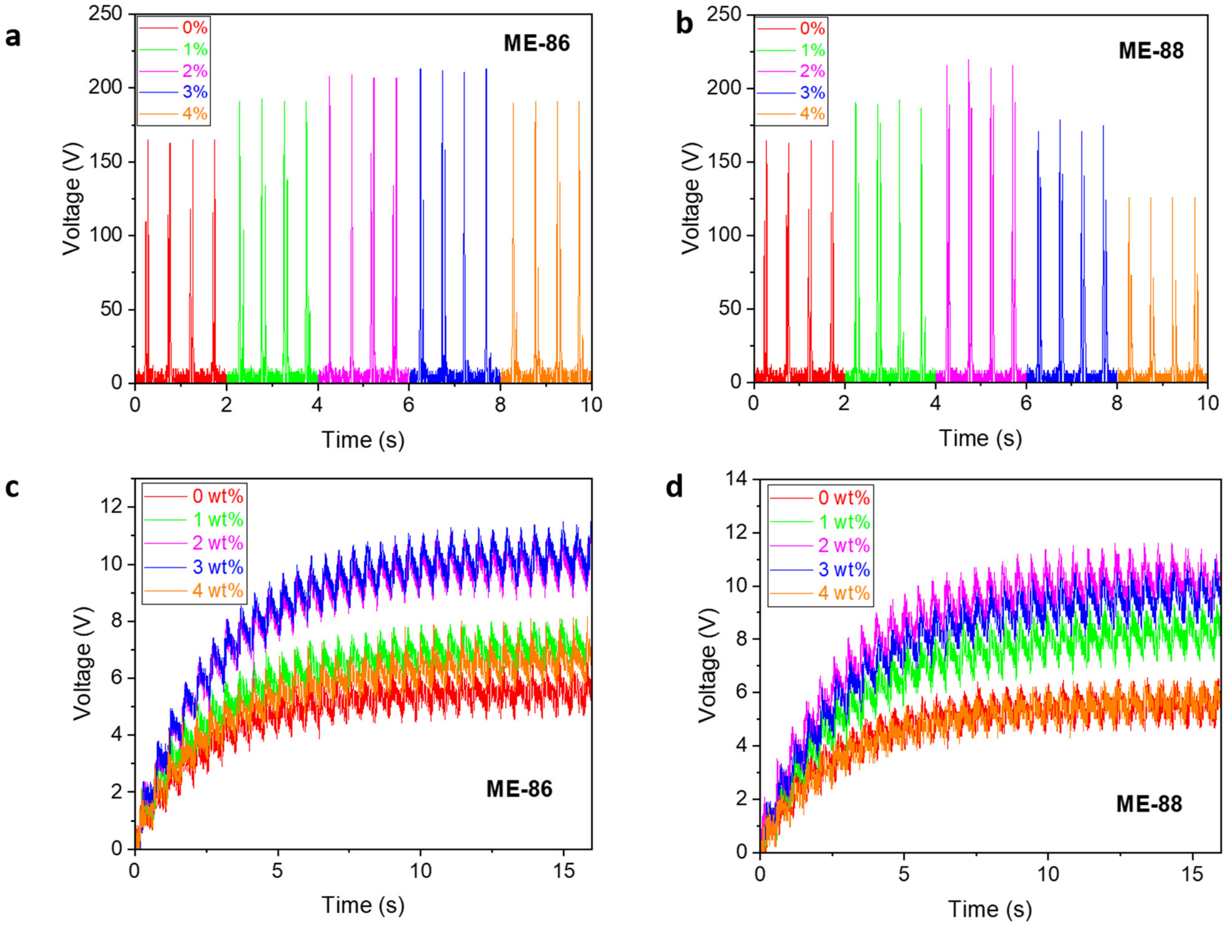


***Figure S10:*** *Instant output voltages and capacitive voltages of ME-86 (a,c) and ME-88 (b,d) doped TENG devices.*

The instant voltages of the devices fabricated from the undoped Nylon and doped PAN are represented in Fig. S10 (a,b). As a reminder, the maximum output voltage of pure PAN has been found as 165V. Also, doping of 1, 2, 3, and 4% fluorocarbon-modified silica have resulted in an increase in the voltage values to 193, 209, 213, and 191V for ME-86, moreover, 193, 220, 179, and 126 V for ME-88. ME-86 has reached the maximum voltage of 213 V at a 3% doping ratio while ME-88 has given a maximum voltage of 220 V at a 2% doping. As can be seen, ME-88 doping shows better performance than ME-86 doping even with less amount of dopant ratio.

Considering the information in the literature, prior studies have noted the destructive effects of air traps and cavities on charge polarization. In the present work, SEM images of the ME-86 and ME-88 indicate that there is an increase in the formation of the node points of the fibers relying on the dopant ratio and it becomes more prominent when the dopant ratio reached 3% for ME-86 and 2% for ME-88. The increase in the amount of dopant produced more cavities and air traps that could adversely affect the charge generation. Also, the study of Bhadra and Siu showed that a smaller chain length is more favorable to produce tight molecular packaging on the surface [3]. Therefore, although both fluorocarbon chains have the same anchoring group and similar chemical structure, the shorter chain on the ME-88 could create better surface coverage and more $Si_{(Silica)}$-O-Si bond on the surface that enhance the tribonegative character of silica particles [4].

Taken together, the charging characteristics of ME-86 and ME-88 are illustrated in Fig. S10 (c,d). A comparison of the best devices (3% ME-86 and 2% ME-88) with the reference one shows that the doping of ME-86 resulted in an increase in capacitive voltage by 75.6% and found as 11.5V that is attributed to 3.39nC stored amount of charge whereas ME-88 has led to the enhancement of capacitive voltage by 77.1% and measured as 11.6 that is corresponding to 3.48nC stored amount of charge. Similar to ME-103 and ME-111, the measured instant output voltage and the calculated highest capacitance voltages are consistent with each other.

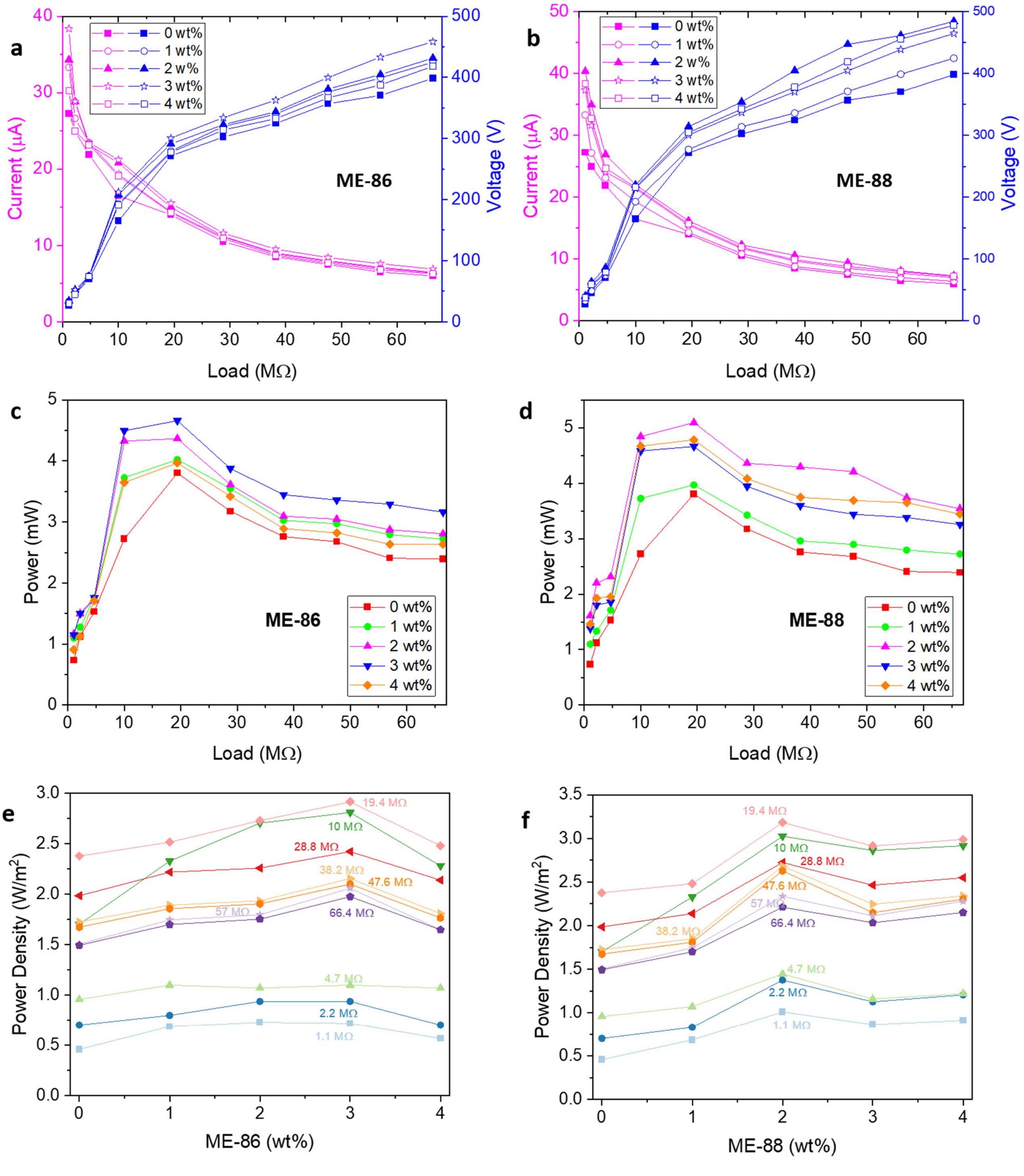


***Figure S11:*** *Power (a,b), Current-Voltage (c,d), and Power Density (e,f) of ME-86 and ME-88 doped TENG devices in different loads.*

Fig. S11 shows the current-voltage and power behavior of ME-86 and ME-88 under altering load. Similar to the ME-103 and ME-111, an increase in the applied loads inversely affects the output current due to the ohmic loss causing a decrease in output current, and this steady decrease continues until the applied load reached 4.7 MΩ. On the other hand, voltage has risen up to the maximum stress point due to the small effect of load up to this point, but surpassing this point has created the opposite effect that led to a decrease in voltage. In comparison to the reference device, the best current and voltage values have been obtained at 1.1 MΩ and 66.4 MΩ, and doping of ME-86 and ME-88 has given rise to a 40 and 15% increase in current (38.4 μA) and voltage (458.2 V) values for ME-86 as well as a 48 and 22% increase (40.36 μA and 484.7 V) for ME-88. The results further support the idea of steric hindrance-regulated surface grafting of molecules in which a smaller alkyl chain improved surface coverage and create more $Si_{(Silica)}$-O-Si bonds making the surface more tribonegative [4].

Output powers of the ME-86 and ME-88 are one of the most well-known tools for assessing dopant contribution to device performance. The best output power and power density of ME-86 were found for 3% doping as 4.7 mW and 2.9 $w/m^2$ at 19.4 MΩ whereas those were 5.1 mW and 3.2 $W/m^2$ for ME-88 under the same load at 2% doping. Variations in the power curves under changing loads are analogous and decrease after they reach maximum power points. These findings support our hypothesis that charge density on the metal oxide can be regulated or balanced in favor of desired triboelectric features.


1. Alptekin, H.; Arkan, E.; Özbek, C.; Can, M.; Farzaneh, A.; Sütçü, M.; Okur, S.; Cobley, A. J., Water affinity guided tunable superhydrophobicity and optimized wettability of selected natural minerals. *Journal of Coatings Technology and Research* **2019,** 16, 199-211.
2. Firdous, I.; Fahim, M.; Daoud, W. A., Performance enhancement of triboelectric nanogenerator through hole and electron blocking layers-based interfacial design. *Nano Energy* **2021,** 82, 105694.
3. Bhadra, P.; Siu, S. W., Comparison of biomolecular force fields for alkanethiol self-assembled monolayer simulations. *The Journal of Physical Chemistry C* **2017,** 121, (47), 26340-26349.
4. Tripp, C.; Veregin, R.; McDougall, M.; Osmond, D., Reaction of alkylchlorosilanes with silica: Importance of a surface attachment in defining the triboelectrification of the modified silica. *Langmuir* **1995,** 11, (6), 1858-1859.
